# Survey on worldwide trauma team activation requirement

**DOI:** 10.1007/s00068-020-01334-z

**Published:** 2020-03-02

**Authors:** Christian Waydhas, Heiko Trentzsch, Timothy C. Hardcastle, Kai Oliver Jensen, the World-Trauma TAcTIC Study Group, Khaled Tolba Younes Abdelmotaleb, George Abi Saad, Markus Baacke, Nehat Baftiu, Christos Bartsokas, Lars Becker, Marco Luigi Maria Berlusconi, Artem Bespalenko, Dan Bieler, Martin Brand, Edilson Carvalho de Sousa Júnior, Narain Chotirosniramit, Yuhsuan Chung, Lesley Crichton, Peter De Paepe, Agron Dogjani, Dietrich Doll, Ayene Gebremicheal Molla, Timothy C. Hardcastle, Timothy C. Hardcastle, Kastriot Haxhirexha, Kajal Jain, Kai Oliver Jensen, Andrey Korolev, Li Zhanfei, Jerry K. T. Lim, Fredrik Linder, Nurhayati Lubis, Nina Magnitskaya, Damian MacDonald, Martin Mauser, Gerrit Matthes, Kimani Mbugua, Sergey Mlyavykh, Barbaro Monzon, Munkhsaikhan Togtmol, Khreshi Mustafa, Michael Mwandri, Pradeep Navsaria, Stefan Nijs, Francisco Olmedo, Maria C. Ortega Gonzalez, Jesús Palacios Fantilli, Marinis Pirpiris, Francois Pitance, Eoghan Pomeroy, M. A. Sadakah, Tapas Kumar Sahoo, Iurie Saratila, Sandro Scarpelini, Uwe Schweigkofler, Edvin Selmani, Tim Søderlund, Michael Stein, Buland Thapa, Heiko Trentzsch, Teodora Sorana Truta, Selman Uranues, Christian Waydhas, Christoph G. Wölfl, Sandar Thein Yi, Ihor Yovenko, Pablo Zapattini

**Affiliations:** 1 Berufsgenossenschaftliches Universitätsklinikum Bergmannsheil, Bürkle-de-la-Camp-Platz 1, 44789 Bochum, Germany; 2 Medical Faculty of the University Duisburg-Essen, University Hospital, Hufelandstr. 55, 45147 Essen, Germany; 3 Institut für Notfallmedizin und Medizinmanagement (INM), Klinikum Der Universität München, Ludwig-Maximilians-Universität, Schillerstr. 53, 80336 Minich, Germany; 4 Committee On Emergency Medicine, Intensive Care and Trauma Management (Sektion NIS) of the German Trauma Society, Berlin, Germany; 5 Inkosi Albert Luthuli Central Hospital, Mayville and University of Kwa Zulu Natal, 800 Vusi Mzimela Rd, Congella, 4058 South Africa; 6 Klinik für Traumatologie, UniversitätsSpital Zürich, Rämistrasse 100, 8091 Zurich, Switzerland

**Keywords:** Trauma team, Trauma team activation, Field triage, Overtriage

## Abstract

**Purpose:**

Trauma team activation (TTA) is thought to be essential for advanced and specialized care of very severely injured patients. However, non-specific TTA criteria may result in overtriage that consumes valuable resources or endanger patients in need of TTA secondary to undertriage. Consequently, criterion standard definitions to calculate the accuracy of the various TTA protocols are required for research and quality assurance purposes. Recently, several groups suggested a list of conditions when a trauma team is considered to be essential in the initial care in the emergency room. The objective of the survey was to post hoc identify trauma-related conditions that are thought to require a specialized trauma team that may be widely accepted, independent from the country’s income level.

**Methods:**

A set of questions was developed, centered around the level of agreement with the proposed post hoc criteria to define adequate trauma team activation. The participants gave feedback before they answered the survey to improve the quality of the questions. The finalized survey was conducted using an online tool and a word form. The income per capita of a country was rated according to the World Bank Country and Lending groups.

**Results:**

The return rate was 76% with a total of 37 countries participating. The agreement with the proposed criteria to define post hoc correct requirements for trauma team activation was more than 75% for 12 of the 20 criteria. The rate of disagreement was low and varied between zero and 13%. The level of agreement was independent from the country’s level of income.

**Conclusions:**

The agreement on criteria to post hoc define correct requirements for trauma team activation appears high and it may be concluded that the proposed criteria could be useful for most countries, independent from their level of income. Nevertheless, more discussions on an international level appear to be warranted to achieve a full consensus to define a universal set of criteria that will allow for quality assessment of over- and undertriage of trauma team activation as well as for the validation of field triage criteria for the most severely injured patients worldwide.

**Electronic supplementary material:**

The online version of this article (10.1007/s00068-020-01334-z) contains supplementary material, which is available to authorized users.

## Introduction

Major trauma is one of the leading causes of death all over the world [1]. To improve basic trauma care worldwide, a trauma checklist has been suggested by the WHO [2]. Advanced trauma care requires a pre-hospital rescue system and a network of trauma centers so that the right patient will be brought to the right initial medical care or can be transferred to the right hospital in the least possible time.

Trauma team activation (TTA) is thought to be essential for the delivery of advanced and specialized care to very severely injured patients. Field triage is intended to allow pre-hospital emergency medical care providers to identify such patients and to trigger TTA at an early time point to get personal and equipment at the resuscitation room ready—at best before the arrival of the patient at the hospital [3].

However, these instruments have been the focus of an ongoing debate regarding over- and under-utilization of hospital resources [4–6], because the more sensitive the TTA criteria are, the more overtriage (i.e. number of patients that fulfill TTA criteria without having a true medical need) results and the higher the likelihood that the trauma team is activated for patients that neither require nor benefit from the activation. On the other hand, triage criteria with low sensitivity and low specificity may produce considerable undertriage by missing patients who urgently require being treated by a trauma team, but who were not identified [7–11]. Undertriage is undesirable because it may result in avoidable death and morbidity. Thus, it seems sensible to monitor the accuracy of TTA criteria [6]. Up to the present, a wide and largely varying composition of criteria and definitions have been used to describe correct TTA between studies or to calculate undertriage [12–16]. To suggest a generally acceptable standard to identify patients who, in retrospect, will have or would have correctly benefited from TTA a list of 20 items has been proposed by traumatologists in Germany and Switzerland in a previous publication [17]. They could be used as a gold standard by which to establish the ‘accuracy’ of field triage criteria in identifying patients who benefitted from highest level trauma team activation*.* It has to be emphasized that these criteria are intended as an academic study tool to retrospectively classify patients into those who may have benefitted from TTA and those who may not have. At this stage, this is not an attempt to modify field triage criteria.

These criteria have been developed from the viewpoint of countries with highly developed pre-hospital and hospital trauma systems and the universal validity of these parameters has been challenged by the argument that highly developed trauma systems may not be available in middle- and low-income countries [18, 19]. As a matter of fact, there may be significant differences between countries with different income levels as well as within such countries. These may comprise the availability and organization of a pre-hospital rescue system, the possibility and organization of prenotification of a patient to a hospital, the comprehensive coverage by trauma teams within a hospital and the composition of such a trauma team.

Thus, the suggested list of 20 items may not be generally valid in a more global context. To validate these criteria, we initiated an international survey in high-, middle- and low-income countries. The objective of the survey was to identify trauma-related conditions that are thought to require a specialized trauma team in the different settings that may be widely accepted independent of the country’s income level.

## Material and methods

The research question was addressed with a cross-sectional survey design using a web-based and paper-based questionnaire. Potential participants were first contacted by email and asked whether they would agree to participate in the survey. The link to the web-based form as well as a print-out form were sent by email to only those participants who had agreed to participate. By answering the questionnaire, participants gave their consent for further use of the assessed data. The survey responses were not anonymized in the data bank. However, every effort was made not to allow the identification of a participant from the information given in the manuscript. Since the survey did not involve interventions or clinical or patient data, no institutional review board approval was required. The survey was conducted from December 2018 to March 2019. Participating countries/physicians were selected on the basis of personal acquaintances of core group members with physicians entrusted with the care of the seriously injured in these countries as well as personal recommendations of other participants in the survey. The survey participants could be surgeons, trauma surgeons, anesthetists or other specialists involved in the acute care of severely injured patients. For some countries, it could be more than one participant per country. We aimed at a similar distribution between high-, upper-middle-, lower-middle- and low-income countries. Our primary intention was to achieve a high rate of responses rather than as many responders as possible. If we received more than one answer from the same country, we chose to keep all responses in the analysis. The variety of the answers from one country was usually so large that it appeared to reflect different local experiences indicating that in those countries there may not be one universal system in the care of the severely injured.

The questionnaire was constructed based around the proposed 20 standard criteria of correct TTA as suggested in the previous work of the authors [17] (question 12 of the questionnaire, see additional electronic material). Each item could be graded using a three-level rating scale (I agree—I partly agree—I disagree) or denoted as not to be relevant or applicable to one’s setting.

Since the availability, resources and organization of trauma care may significantly differ in the participating countries, additional questions were included in the questionnaire relating to the composition and the availability of trauma teams, the number and qualifications of the trauma team members and data on the equipment in the trauma bay (questions 1, 3–11). Furthermore, information was gathered about the pre-hospital rescue system and the selection of patients due to insurance status within the respective countries (questions 2–2E). Lastly, we assumed that many of the participants of the survey would work predominately in high-level institutions within their countries. Therefore, we also aimed at information regarding the structural facilities in (presumably) lower levels of trauma hospitals within their respective countries (questions 13–20).

The survey was first developed and discussed in two Delphi rounds by the core group of the study. Then, this version of the survey was sent to the participants and they were asked to give feedback on the questionnaire of the survey (e.g. are the questions understandable, are they adequate for the respective setting, are important aspects missing, etc.) and to suggest modifications, improvements or additions. The incoming suggestions for improvement and amendments have been then implemented to finalize the survey questions. Most of the questions and definitions appeared to be self-explanatory and generally applicable. However, two definitions require some explanation. First, the level of a trauma hospital may be denominated differently in different countries. To overcome this, we offered alternative terms that we thought will allow for a comparable classification in the participating countries (e.g. “tertiary or major trauma center/supraregional/highest level” versus “regional trauma center/intermediate level” versus “local or district trauma center/basic level”).

Concerning the type of surgeon who may be part of the trauma team, there may be considerable differences, particularly with respect how to define a trauma surgeon. Therefore, we offered several possibilities to cover the different definitions: Two types of “trauma surgeons”: a general surgeon with extra training in trauma, similar to USA or an orthopedic surgeon with extra training in trauma, like in some European countries. Furthermore, it was possible to choose emergency physician, general or visceral surgeon or orthopedic surgeon.

The online version of the questionnaire was programmed in Google Forms, the print-out form was created in Microsoft Word^®^. The answers were exported to an Excel^®^ (Microsoft Corp, Redmond, USA) sheet via a CSV file (from the online survey) or manually filled into the Excel^®^ file (from the Word questionnaires). The figures were created using Excel^®^.

The classification of the country income level followed the World Bank Country and Lending Groups based on the 2017 data [20]. Low-income countries were defined as countries with a gross national income (GNI) per capita, calculated using the World Bank Atlas method, of $995 or less. Lower-middle-income economies was assumed with a GNI per capita of $996 and $3895. Upper-middle-income economies are those with a GNI per capita between $3896 and $12,055. High-income economies are those with a GNI per capita of $12,056 or more.

## Results

The survey was sent to physicians in 49 countries with a return rate of 76% (*N* = 37). Overall, 69 colleagues have been contacted and 51 eventually participated in the survey (74%). The distribution of their country’s income level is detailed in Table 1.

**Table 1:** Responses according to country income level

	Low-income-countries	Lower-middle-income countries	Upper-middle-income countries	High-income countries
Total number of countries with income level worldwide according to [1]	34	47	56	81
Participating countries	3 (9%)	10 (21%)	10 (18%)	14 (17%)
	Ethiopia Nepal Tanzania	Egypt India Kenya Kosovo Moldova Mongolia Myanmar Palestine Ukraine Zambia	Albania Brazil China Lebanon North Macedonia Paraguay Romania Russian Federation South Africa Thailand	Australia
				Austria
				Belgium
				Canada
				Finland
				Greece
				Ireland
				Israel
				Italy
				Singapore
				Sweden Switzerland Taiwan United Kingdom
Participants	3	13	19	16

Percentage is based on the number of countries which participated in relation to the total number of counties with the same income level

The description of the participating trauma hospitals and the setting in which they work are shown in Table 2. At least two-thirds of the participants classified themselves as of the highest level of care or tertiary care centers, irrespective of the country’s income level. Several regional trauma hospitals and a few local trauma hospitals also participated. The population they served covered the whole range from less than 500,000 to more than 5 million inhabitants. Sixty percent of participating hospitals reported to be the only hospital of this level in their area or city, while 18% were the only hospital of this level within their country. The higher the country’s income level was, the more hospitals were served by an organized pre-hospital rescue system and also more of the severely injured were brought to the hospital by professional ambulances. The vast majority of hospitals had a specially equipped trauma room available and provided a trauma team with the lower-middle-income countries displaying somewhat lower counts. The rate of countries having adopted a system of trauma centers was highest in the middle-income countries.

**Table 2:** Description of the participating trauma hospitals and the settings in which they work. The classification of a trauma center in a country without formal trauma center accreditation was graded by self-assessment. Percentages are calculated within columns

	Overall	Low-income-countries	Lower-middle-income countries	Upper-middle-income countries	High-income countries
Participants	51	3	13	19	16
Level of trauma center					
Tertiary care	38 (75%)	2 (67%)	9 (69%)	16 (84%)	11 (68%)
Regional	10	1	3	2	4
Local/basic	3	–	1	1	1
Not reported	–	–	–	–	–
Population covered per trauma center					
< 500.000	7 (14%)	–	2	3	2
500.000–1 Mio	14 (27%)	–	4	3	7
1–5 Mio	23 (45%)	1	5	11	6
> 5 Mio	7 (14%)	2	2	2	1
Organized pre-hospital rescue system in the country or parts of it					
Yes	46 (90%)	1 (33%)	10 (77%)	19 (100%)	16 (100%)
No	5	2	3	–	–
Not reported	–	–	–	–	–
Brought to hospital by ambulance					
> 75%	31 (61%)	0	4	13	14
50–75%	6 (12%)	0	3	2	1
25–49%	6 (12%)	1	2	3	–
< 25%	4 (8%)	2	–	1	1
Not reported	4 (8%)	–	4	–	–
Concept of trauma centers existing					
Yes	34 (67%)	1 (33%)	5 (39%)	17 (90%)	11 (67%)
No	17 (33%)	2	8	2	5
Not reported	–	–	–	–	
If yes					
Accreditation by government	13	–	2	5	6
Accreditation by independent body	8	–	1	6	2
No accreditation	12	1	2	6	3
Not reported	–	–	–	–	–
Participant’s facility is the only hospital of this level within country					
Yes	9 (18%)	2	3	4	–
No	42 (82%)	1	10	15	16
Not reported	–	–	–	–	–
Participant’s facility is the only hospital of this level within city					
Yes	31 (61%)	3	11	9	8
No	20 (39%)	–	2	10	8
Not reported	–	–	–	–	–
Trauma team available					
Yes	42 (82%)	2	9	17	14
No	9 (18%)	1	4	2	2
Not reported	–	–	–	–	–
Special resuscitation room/area available					
Yes	46 (90%)	3	9	18	16
No	5 (10%)	–	4	1	–
Not reported	–	–	–	–	–
Hospital treatment free of charge or covered by insurance in > 90% of patients					
Yes	36 (71%)	1	9	11	15
No	15 (29%)	2	4	8	1
Not reported	–	–	–	–	–

The agreement with the proposed parameters and conditions for post hoc identification of a situation that would have required a trauma team for the initial care of patients is shown in Table 3. The highest rate of full agreement was observed for “Glasgow coma scale < 9″ (90%) and “respiratory rate < 9 or > 29/min” (90%). The rate of full agreement was more than 75% for “pericardiocentesis”, “advanced airway management”, “pulse oximetry (SpO2) < 90%”, “emergency surgery”, “systolic blood pressure < 90 mmHg”, “shock index > 0.9”, “cardiopulmonary resuscitation”, “deterioration of GCS ≥ 2 points before admission”,” chest tube or needle decompression” and “catecholamine administration”. Eight conditions did not achieve the 75% full agreement level, although their rate of partial agreement was high (12–31%). Overall, the highest rate of agreement was found for abnormal vital functions (respiratory, cardio-circulatory and cerebral dysfunction) and for a number of invasive life-saving procedures to counteract such disturbances. Outcome-related criteria (ICU treatment, death) were rated as less relevant.

**Table 3:** Percentage of agreement with criterion (cases not reported are excluded), ordered by magnitude of full agreement

TTA criterion	Full agreement	Partial agreement	Disagreement	Not relevant	Not reported
Glasgow Coma Scale (GCS) < 9	90% (46)	8% (4)	–	2% (1)	–
Respiratory rate < 9 or > 29/min	90% (46)	8% (4)	–	2% (1)	–
Pericardiocentesis	88% (43)	6% (3)	2% (1)	4% (2)	2
Advanced airway management	86% (44)	12% (6)	–	2% (1)	–
Pulse oximetry (SpO2) < 90%	86% (44)	12% (6)	–	2% (1)	–
Vascular, neurosurgical, abdominal, thoracic, pelvic, spinal or extremity-saving surgery^#^	84% (42)	12% (6)	2% (1)	2% (1)	1
Systolic blood pressure < 90 mmHg	82% (42)	16% (8)	–	2% (1)	–
Shock index > 0.9	82% (40)	16% (8)	–	2% (1)	2
Cardiopulmonary resuscitation	80% (41)	14% (7)	4% (2)	2% (1)	–
Deterioration of GCS ≥ 2 points before admission	80% (40)	16% (8)	2% (1)	2% (1)	1
Chest tube or needle decompression	78% (40)	18% (9)	4% (2)	–	–
Catecholamine administration	76% (38)	20% (10)	2% (1)	2% (1)	1
Death within 24 h	73% (35)	12% (6)	13% (6)	2% (1)	3
Hypothermia < 35°	72% (36)	20% (10)	4% (2)	4% (2)	1
> 2 external fixators (humerus, femur, pelvis)	72% (35)	20% (10)	4% (2)	4% (2)	2
Abbreviated injury scale (AIS) ≥ 4	70% (35)	24% (12)	2% (1)	4% (2)	1
ICU length of stay > 24 h	62% (31)	26% (13)	8% (4)	4% (2)	1
Transfusion	61% (31)	31% (16)	6% (3)	2% (1)	-
Tourniquet (pre-hospital)	60% (30)	28% (14)	6% (3)	6% (3)	1
Radiological therapeutic intervention^§^	60% (29)	28% (14)	6% (3)	6% (3)	2

Number of participants in parenthesis

Generally speaking, the rate of disagreement with the proposed criteria was very low. In particular, the rate of disagreement was zero for “Glasgow coma scale < 9”, “respiratory rate < 9 or > 29/min”, “advanced airway management”, “pulse oximetry (SpO2) < 90%”, “systolic blood pressure < 90 mmHg” and “shock index > 0.9”. Disagreement was also low (below 5%) for most of the other criteria, except for “transfusion” (6%), “pre-hospital use of a tourniquet” (6%), “radiological therapeutic intervention” (6%), “ICU length of stay > 24 h” (8%) and “death within 24 h” (13%). There was no difference between participants with disagreement with respect to income level (5 low- and lower-middle- vs. 5 upper-middle- and high-income countries), with a trend towards more regional trauma hospitals (6 tertiary vs. 4 regional hospitals) and those without a trauma team (7 with vs. 3 without trauma team).

Only very few respondents indicated that a specific criterion was not relevant to their setting. These participants originated from high-income countries (*N* = 1), upper-middle-income countries (*N* = 3), lower-middle-income countries (*N* = 2) or low-income countries (*N* = 1) and reported for tertiary care hospitals (*N* = 6) or regional hospitals (*N* = 2).

This leaves a rate of partial agreement for the criteria ranging from 6 to 31%. “Abbreviated injury scale (AIS) ≥ 4”, “ICU length of stay > 24 h”, “Transfusion”, “Tourniquet (pre-hospital)” and “Radiological therapeutic intervention” showed the highest rate of partial agreement of more than 25% of participants.

**Fig. 1:**
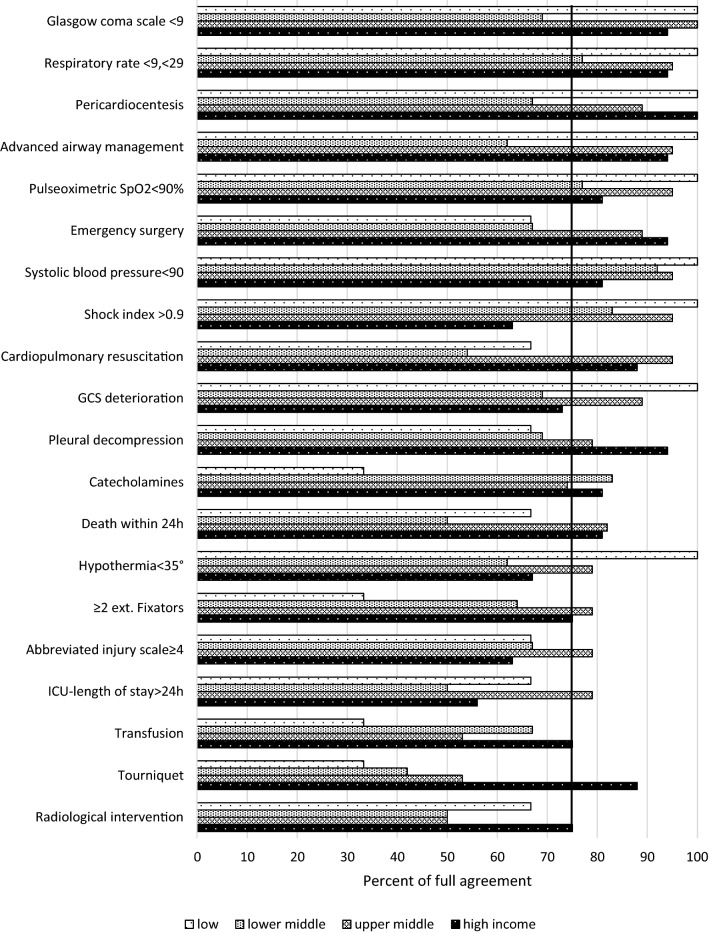
Distribution of full agreement with criterion in relation to the countries’ income level. The vertical line denominates 75%—the level of agreement

**Fig. 2:**
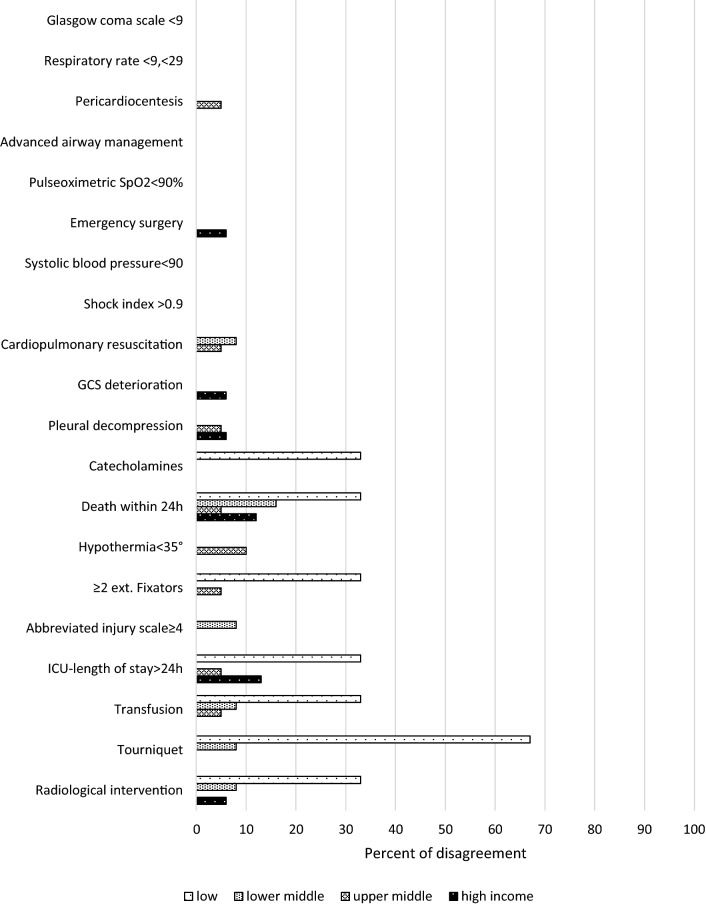
Distribution of disagreement with criterion in relation to the countries’ income level

The level of full agreement and of disagreement in the different income levels is shown in Figs. 1 and 2, respectively. The level of full agreement (Fig. 1) was highest in the high and upper-middle-income countries with the majority of criteria reaching 75% or more of full agreement. Some observations deserve mentioning. In high-income countries “shock index > 0.9”, “abbreviated injury scale ≥ 4”, “hypothermia < 35°” and “ICU length of stay > 24 h” displayed a lower rate of full agreement, lower than in the upper-middle-income countries and in the same range as the lower-middle-income countries. “Pleural decompression”, “transfusion”, “tourniquet use” and “radiological therapeutic intervention” received less full agreement in the upper-middle-, lower-middle- and low-income countries as compared to high-income countries. In general, the rate of full agreement was lowest in the lower-middle-income countries with the exception of “catecholamine administration” and “transfusion” and the low-income countries. It is of note that all three participants of low-income countries fully agreed on all of the criteria of abnormal vital signs (cerebral, respiratory, cardiocirculatory, hypothermia).

The rate of disagreement (Fig. 2) was below 10% independent of the countries’ income level with a few exceptions: “death within 24 h” (12%) and “ICU length of stay > 24 h” (13%) in high-income countries and “death within 24 h” (16%) in lower-middle-income countries.

For seven criteria, one or two (out of three) participants of the low-income countries disagreed. In the upper-middle-income countries, the rate of disagreement was 10% or lower with all criteria.

## Discussion

There was a high rate of full agreement with the suggested criteria to be used for the post hoc definition of the requirement for trauma team activation of at least 75% with 12 of the proposed criteria. These included “Glasgow coma scale < 9”, “respiratory rate < 9 or > 29/min”, “pericardiocentesis”, “advanced airway management”, “pulse oximetry (SpO2) < 90%”, “emergency surgery”, “systolic blood pressure < 90 mmHg”, “shock index > 0.9”, “cardiopulmonary resuscitation”, “deterioration of GCS ≥ 2 points before admission”,” chest tube or needle decompression” and “catecholamine administration”. They comprised nearly all of the criteria of abnormal vital signs and most of the criteria of life-saving interventions. This level of agreement was similar to the threshold of agreement of 75% and 80%, respectively, that had to be achieved in two different consensus statements for a criterion to be included [15, 17]

Looking at the level of full agreement, however, revealed some potential differences in the evaluation of some of the criteria with respect to a country’s income level. Interestingly, some of the criteria reached a lower level of full agreement in the high-income countries than in the upper-middle-income counties. The level of full agreement tended to be lower in the lower-middle- as compared to the upper-middle-income countries. The number of participants in the low-income countries was low so that the results could be highly variable. The rate of full agreement for criteria of abnormal vital signs (cerebral, respiratory, cardio-circulatory, hypothermia) as well as of advanced airway management tended to highest in the low-, upper-middle- and high-income countries in contrast to the lower-middle-income countries.

On the other hand, the level of disagreement was rather low, mostly expressed by single participants. The highest rate of disagreement (> 5%) was observed for “death within 24 h”. This leaves a number of the proposed criteria with partial agreement, i.e. a level of less than 75% of full agreement but less than 5% disagreement (“hypothermia < 35°”, “ > 2 external fixators (humerus, femur, pelvis), “abbreviated injury scale (AIS) ≥ 4”, “ICU length of stay > 24 h”, “transfusion”, “tourniquet (pre-hospital)” and “radiological therapeutic intervention”.

Although the general full agreement for the proposed criteria of post hoc trauma team requirement is very high irrespective of the country’s income level, some of the criteria may deserve some more discussion before they may reach a higher level of agreement (or are being rejected). There is no uniform pattern of different levels of agreement in the order of a country’s income. There is less full agreement for some of the criteria in high- versus upper-middle-income countries.

The survey was like a single-point vote without the possibility of discussing the different items with the other participants. Therefore, partial agreements might be “upgraded” to full agreement (or disagreed) after the exchange of arguments and rationales. This would be the normal process of achieving consensus. In the cited consensus statements, up to five voting rounds were required to achieve agreement [15, 17]. Having this in mind, the agreement within this single survey voting appears high and it may be concluded that the proposed criteria may be useful for most countries independent of their level of income. Nevertheless, more discussions on an international level appear to be warranted to achieve a full consensus to define a universal set of criteria that will allow for quality assessment of over- and undertriage of trauma team activation for the most severely injured patients worldwide.

The major limitation of the study is the selection bias introduced by selecting the participants based on personal knowledge and recommendation. The participants were contacted based on the recommendation of 12 different members of the study group. They may lack the possible variability of opinions compared to a random sample. Therefore, the selection of participants appears arbitrary and not representative. For example, there is a preponderance of tertiary care hospitals and the low number of hospitals included from low-income countries may bias interpretation of this proportion of the study cohort. On the other hand, our participants represent different disciplines involved in trauma care such as trauma surgeons (general surgeons), trauma surgeons (orthopedic surgeons), anesthesiologists or emergency physicians thus representing a variety of different backgrounds. Also, a high rate of responders and the personal acquaintance may offer the chance to receive valid and thoughtful answers, particularly concerning the answers about the trauma team requirement, where some thorough thought on the side of the participants is essential. Other types of selection may introduce other biases. The variation of using an “official” mailing list from worldwide active organizations would have resulted in a more representative list of countries. However, it remains arbitrary who of the physicians contacted would actually answer, which may reduce the representativeness. The less personal character of the contact with the potential participant may also confound the thoughtfulness of some of the answers. Our return rate of 75.5% compares favorably to the return rate of a study using mailing lists from international societies and networks (54%) [21]. Including participants from one medical society (e.g. only surgeons) may also bias the results. In both approaches, it is not clear whether the answers would be representative for a whole country or would be more specific to the institution and the setting of the respondent. That this may be the case was shown by the quite differing answers from participants originating in the same country. Bearing this in mind, our results have to be interpreted with caution.

However, many of the answers received in the survey of Miclau et al. [21] and our study are quite comparable: There was a similar availability of designated trauma centers (33.3 vs. 27.8% in low-income countries and 68.8 vs. 71.0% in high-income countries). The availability of a formalized emergency medical service is in the same range for all levels of income in both studies, indicating that the participating countries in our survey may not substantially differ from a larger cohort of countries. In a systematic review about trauma systems around the world 32 countries have been evaluated [22]. The authors included fewer low- and middle-income countries (*N* = 9) compared to 23 countries of that type in our study. Therefore, their results with respect to tertiary care trauma centers and the availability of a trauma team may not be comparable to ours. Furthermore, 84% of the publications used in their study were older than 5 years and 50% older than 10 years, so that considerable improvements may have taken place since then in many countries.

There was a preponderance of participants from tertiary care hospitals in all country income levels. Their experience and rating may be different from physicians working in regional or local hospitals. It might be speculated that such differences could be more pronounced in countries with a lower per capita income [21]. However, the assessment from our participants from regional or local trauma hospitals did not differ substantially from those from tertiary care hospitals, although the numbers are too small to rule out this possibility. To get a definite answer, it would require interviewing physicians from hospitals from all levels of trauma care in each country. Nevertheless, it appears reasonable to assume that the participants of our study did not answer in complete contradiction to the general rating within their respective country.

The number of participating countries in our survey amounted to around 20% of all countries within the same income level, with a clear underrepresentation of low-income countries. Therefore, the high level of agreement shown in our survey may not be true for low-income countries. Indeed, Miclau et al. [21] have shown, that even in designated trauma centers in low-income countries, important musculoskeletal injury resources such as spine board, pelvic binder, computed tomography or post-anesthesia care unit are lacking to a much higher degree in comparison with countries with lower-middle-income or higher-income level.

Although we have gathered information about pre-hospital trauma care and facility-based trauma care, we cannot assign to our participants the WHO trauma maturity index [23], because we are lacking information about education and training and quality assurance. We did not explicitly assess whether our participants in low- and middle-income countries fulfill the Bellwether procedures for essential surgical care like cesarean delivery, laparotomy and treatment of open fractures [24]. Since all of them do have a general surgeon (100%), a trauma or orthopedic surgeon (100%) and a gynecologist (69%) as well as a specially equipped resuscitation area available, they could be classified as fulfilling the requirements of a high-level care.

Despite these limitations, it appears valid to assume that the proposed criteria for correct trauma team activation may be useful not only for high-income countries but at least also for lower-middle- and upper-middle-income countries. Although the requirements they pose may not be met in low-income countries and the entire territory of middle-income countries they appear to be recognized by many of the physicians practicing in these countries as well as in the high-income countries. They could be used for quality assurance in the care of severely or polytraumatized patients within trauma hospitals with benchmarks individualized by countries and dynamic over time. While there appears to be a large subset of criteria with high universal acceptance, some of the criteria with only partial agreement or even disagreement will have to be discussed in the future on a worldwide or a country-specific level to recommend which patient should or should not have received trauma team activation. A generally accepted or locally adapted criterion standard could be used to validate field triage criteria as well as to measure the performance of trauma systems in the different countries and adapt them to their specific conditions, circumstances and resources. It could further be used to compare the efficiency and capabilities of the initial care of severely injured patients worldwide.

## Electronic supplementary material

Below is the link to the electronic supplementary material.

Supplementary file1 (DOCX 49 kb)
