# Supplementary material for: Survey on worldwide trauma team activation requirement
Source: Eur J Trauma Emerg Surg. 2020 Mar 2;47(5):1569–80. doi: 10.1007/s00068-020-01334-z (PMC8476357; doi:10.1007/s00068-020-01334-z)
Supplement: Supplementary file 1 — Supplementary file1 (DOCX 49 kb) [file 68_2020_1334_MOESM1_ESM.docx]

**Supplemental Material**

Survey on Worldwide Trauma-Team Activation Requirement

**Additional Electronic Material – Survey Form**

Survey on **World**wide **Trauma**-**T**eam **Ac**tivation and **T**rauma/**I**njury **C**are (WORLD- Trauma TAcTIC)

Please, indicate your country:

Please, give your name:

**General Aspects of Trauma Care in your Country**

1. In a general sense, does the concept of “trauma centers” apply to your country’s health care system at all?

Yes

No

**IF YES**: please answer questions 1A and 1B

**IF NO**: please move on to question 2

**1A** How many levels of trauma centers do you have in your country?

1

2

3

4

Unspecified

**1B** Does accreditation as a trauma center in your country follow a protocol or
 certification scheme (in contrast to self-rating)?

Yes

No

**IF YES to 1B** please answer question 1C, otherwise move on to question 2

**1C** Is the accreditation system government led or through an independent body?

Government

Independent body

1. Is there an organized professional prehospital rescue system in your country?

Yes, covering most of the country

Yes, covering only parts of the country (urban and rural)

Yes, covering only parts of the country (mainly urban)

Yes, but only a few selected areas

No

**If any YES**: Please answer questions 2A to 2E, otherwise move on to question 3.

**2A** Please answer for your city/area, if it is not the same for all parts of your
 country where prehospital rescue systems are established.
 I will give the answers to the following questions for

My country

My city / area

**2B** How is the prehospital rescue dispatch system organized?

One dispatch center covering the/a whole city/area

More than one dispatch center covering the/a whole city/area, but
 cooperating and responsible for distinct areas

More than one dispatch center covering the same city/area, not
 cooperating or not matching with each other

Dispatch center(s) with no formal responsibility

Dispatch center run by your own hospital

No dispatch centers

**2C** What types of rescue vehicles are available? Multiple answers are possible.

Patient transport vehicle (= Vehicle to transport a sick or injured person, manned with a single driver without any medical training)

Simple ambulance (= Vehicle to transport a sick or injured person, manned with a single driver with some medical training)

Standard Ambulance (= Vehicle to transport a sick or injured person, manned with a team of at least two persons of whom at least one person has received a specific medical training i.e. emergency medical technician)

Advanced life support ambulance or Response vehicle (=Vehicle to transport a sick or injured person, manned with a team of at least two persons with specific medical training of whom at least one received advanced medical training i.e. paramedic and with advanced medical equipment (e.g. defibrillator, ventilator)

Ambulance or rapid response vehicle with an emergency physician

Air-borne emergency medical service staffed by paramedics or nurses with special training (without on-board emergency physician)

Air-borne emergency medical service with on-board emergency physician

**2D**  Is access to the prehospital rescue system free of charge for residents?

Yes

No

**If NO**: Please answer question 2D-1, otherwise move on to question 2E

**2D-1** How large is the proportion of patients whose payment is covered by
 health insurance (vs. private payment)? Please give an estimate.

>90%

75-90%

50-74%

25-49%

10-24%

<10%

**2E** What percentage of severely injured patients (e.g. ISS>15) are brought to
 your hospital by ambulance (vs. private vehicle transport)?

>75%

50-74%

25-49%

<25%

**For your own center:**

1. How large is the population in the city/area where your trauma center is located?

<500.000

500.000 to 1 Mio

1 Mio to 5 Mio

>5 Mio

1. How would you rate your trauma hospital / trauma center? Please mark the most appropriate answer

Tertiary or Major Trauma Center / supraregional / highest level

Regional Trauma Center / intermediate level

Local or District Trauma Center/ basic level

Other, non-trauma facility

1. Is your hospital the only hospital with this level of care in your country

Yes

No

**If NO**: Please answer question 5A, otherwise move to question 6

**5A**  How many hospitals of your level do you have in your country (please give an estimate)

1. Is your hospital the only hospital with this level of care in your city / area

Yes

No

1. Do you have medical professionals (nurse or physician) seeing the trauma patient immediately on arrival in the emergency department?

Yes

No

**If YES**: please answer question 7A1, otherwise move on to question 7A2

**7A1** What type of medical professional is it?

Nurse

Physician

Other

Move on to question 8

**7A2** How long will it take until a patient with severe injuries will be seen by a physician?

<5min

6 to 10min

10 to 15min

15 to 30min

>30min

1. Do you have a (Trauma) Team at your hospital that, if activated, immediately **comes together at the patient’s bedside in the emergency department** if the patient is or is thought to be severely injured?

Yes

No

**If YES**: Please answer questions 8A and 8B

**If NO**: Please answer questions 8C

**8A** Who is part of the trauma team? More than one answer is possible

Trauma surgeon (general surgeon with extra training in trauma, similar to USA)

Trauma surgeon (orthopedic type of surgeon with extra training in trauma, like some European countries)

Anesthesiologist

Emergency physician

General or visceral surgeon

Orthopedic surgeon

Neurosurgeon

Radiologist

Other specialist physicians

Other non-specialist physicians

Emergency department or surgical nurse

Nurse with specific training in trauma

Anesthesiology nurse

Radiology technician

Physician extenders

Other

**8B** What can lead to trauma team be activation? More than one answer is possible

Advance notice from the prehospital rescue team or dispatch center

Using the American College of Surgeons criteria (or local modification) for trauma team activation

Triage into the highest priority using another triage system (e.g. Manchester triage system, SATS or similar)

At the discretion of the nurse or another (non-doctor) medical professional with first contact

At the discretion of the physician with first contact

other

**8C** Who initially takes care of such a severely injured patient? More than one answer is possible

Trauma surgeon (either of the two types as described above)

General or Visceral Surgeon

Orthopedic surgeon

Anesthesiologist

Emergency physician

Other specialist physicians

Other non-specialist physicians

Emergency department or surgical nurse

nurse with specific training in trauma

Anesthesiology nurse

Physician extenders

Other

1. Do you have a specially equipped resuscitation area / trauma room to care for the severely injured patients?

Yes

No

**If YES**: Please answer questions 9A to 9C, otherwise move on to question 10

**9A** Which patients will be treated in the resuscitation area / trauma room? More than one answer is possible.

All patients with trauma team activation

Not all patients with trauma team activation

Some patients without trauma team activation

Some patients: No trauma team available in the hospital

**9B** Which of the following conditions would lead to a treatment in the
 resuscitation area / trauma room? Do only mark if applicable in your setting.
 More than one answer is possible

Triage Score with highest priority

Hypotension (systolic blood pressure below 90 mmHg)

Prehospital catecholamines

Respiratory distress

SpO_2_ below 90% despite administration of oxygen via mask

Respiratory rate >29 or <9/min

Prehospital intubation/advanced airway

Glasgow Coma Scale < 15

Glasgow Coma Scale < 9

Deterioration of Glasgow Coma Scale of more than 2 points during treatment

Massive external bleeding

Mechanism of injury

Gunshot wounds to the trunk or head/neck

Stab wounds to the trunk or head/neck

Open fracture of a long bone (e.g. femur/tibia)

Hypothermia < 35° C (<95° F)

Prehospital chest tube/needle decompression

CPR

other

**9C** What equipment is held available in the resuscitation area / trauma room?
 More than one answer is possible.

Monitor for vital signs

Defibrillator

Perfusion pumps for catecholamines

Ultrasound machine

Mobile X-ray

CT-scanner

Equipment for tracheal intubation

Ventilator

Equipment for chest tube insertion

Equipment for pericardiocentesis

Equipment for emergency thoracotomy or laparotomy

Blood and blood products

1. Which specialists (or diagnostic tools) do you have available in the hospital within 20min (24/7)? More than one answer is possible.

CT

MRI

General radiologist

Interventional radiologist

Neurosurgeon

ENT-surgeon

Maxillofacial surgeon

Thoracic surgeon

Visceral / General surgeon

Vascular surgeon

Plastic surgeon

Trauma surgeon (either of the two types as described above)

Orthopedic surgeon

Urologist

Ophthalmologist

Pediatrician

Gynecologist

Cardiologist

1. Is emergency medical treatment for trauma patients free of charge for residents at your hospital?

Yes

No

Partly

**If NO or partly**: Please answer question 11A, otherwise move on to question 12

**11A**  How large is the proportion of patients whose payment is covered by health insurance (vs. private payment)?

>90%

75-90%

50-74%

25-49%

10-24%

<10%

**If partly**: Please specify what treatment will be free of charge

1. For which of the following criteria or conditions or treatments would you agree that the activation of a specialized trauma team is required or reasonable or would have been required in retrospect. Since there is no generally accepted composition of a trauma team it remains at your discretion what you consider a trauma team. However, it should be a team of at least one nurse and one physician specialized in trauma care treating the patient in a specially equipped trauma room or resuscitation area and which arrives immediately and without delay at the patient’s bedside after activation and remains with the patient until he or she is transferred to the OR or the ICU or a transfer to the normal ward is decided.

|  | I agree | I partly agree | I disagree | Not relevant or applicable in my setting |
| --- | --- | --- | --- | --- |
| Abbreviated injury scale (AIS) ≥ 4 |  |  |  |  |
| ICU-length of stay > 24 h |  |  |  |  |
| Death within 24 h |  |  |  |  |
| Invasive procedures (pre-hospital or in the emergency room) | | | | |
| Cardiopulmonary resuscitation |  |  |  |  |
| Advanced airway management |  |  |  |  |
| Chest tube or needle decompression |  |  |  |  |
| Pericardiocentesis |  |  |  |  |
| Tourniquet use (pre-hospital) |  |  |  |  |
| Catecholamine administration |  |  |  |  |
| Transfusion |  |  |  |  |
| Surgical/therapeutic radiological intervention | | | | |
| Vascular, neurosurgical, abdominal, thoracic, pelvic, spinal or extremity-sparing surgery |  |  |  |  |
| Radiological therapeutic intervention |  |  |  |  |
| C2 external fixators (humerus, femur, pelvis) |  |  |  |  |
| Abnormal vital signs | | | | |
| Pulse oximetry (SpO2) < 90% |  |  |  |  |
| Respiratory rate <9 or >29/min |  |  |  |  |
| Systolic blood pressure<90 mmHg |  |  |  |  |
| Shock index >0.9 |  |  |  |  |
| Glasgow Coma Scale (GCS) < 9 |  |  |  |  |
| Deterioration of GCS > 2 points before admission |  |  |  |  |
| Hypothermia <35°_ |  |  |  |  |

**In addition to the conditions stated above I suggest that under the following conditions the trauma team should be activated or should have been activated** (please indicate only such conditions that you think would post-hoc require a trauma team. Do not indicate mere field triage criteria.

**Optional:**

**To get an estimate about the treatment of severely injured patients in your country (apart from your own hospital) we ask you to answer the following questions (**to the best of your knowledge). **If appropriate, please give distinct answers for Regional (intermediate level) and Local or District (basic level) (trauma) hospitals.**

**For Regional Trauma Centers / intermediate level**

1. How many (trauma) hospitals of this level do you have in your country (give an estimate)?

1

2 to 5

6 to 10

10 to 25

26 to 50

>50

1. How many of them do have a trauma team?

<5%

6-25%

26-49%

50-75%

>75%

1. How many of them do have a resuscitation area / trauma room?

<5%

6-25%

26-49%

50-75%

>75%

1. What specialists (or diagnostic tools) will be present / available at the majority these hospitals or available within 20 min? More than one answer is possible.

General physician (without specialty)

Emergency physician

Surgical or emergency nurse

General nurse

Anesthesiology nurse

General surgeon

Anesthesiologist

Orthopedic surgeon

Trauma surgeon

CT

MRI

General radiologist

Interventional radiologist

Neurosurgeon

ENT-surgeon

Maxillofacial surgeon

Thoracic surgeon

Vascular surgeon

Plastic surgeon

Urologist

Ophthalmologist

Pediatrician

Gynecologist

Cardiologist

**For Local or District Trauma Center/ basic level**

1. How many (trauma) hospitals of this level do you have in your country (give an estimate)?

1

2 to 5

6 to 10

10 to 25

26 to 50

>50

1. How many of them do have a trauma team?

<5%

6-25%

26-49%

50-75%

>75%

1. How many of them do have a resuscitation area / trauma room?

<5%

6-25%

26-49%

50-75%

>75%

1. What specialists (or diagnostic tools) will be present / available at the majority these hospitals or available within 20 min? More than one answer is possible.

General physician (without specialty)

Emergency physician

Surgical or emergency nurse

General nurse

Anesthesiology nurse

General surgeon

Anesthesiologist

Orthopedic surgeon

Trauma surgeon

CT

MRI

General radiologist

Interventional radiologist

Neurosurgeon

ENT-surgeon

Maxillofacial surgeon

Thoracic surgeon

Vascular surgeon

Plastic surgeon

Urologist

Ophthalmologist

Pediatrician

Gynecologist

Cardiologist
